# Get Back, a person-centered digital program to promote physical activity among patients undergoing spinal stenosis surgery: a randomized feasibility study

**DOI:** 10.1186/s40814-026-01826-6

**Published:** 2026-04-24

**Authors:** Christian Ernest, Rikard Hanafi, Helena Brisby, Andreas Fors, Håkan Hedman, Mike Kemani, Mari Lundberg, Jo Nijs, Hedvig Zetterberg, Emelie Karlsson

**Affiliations:** 1 Back in Motion Research Group, Department of Health Promoting Science, Sophiahemmet University, Stockholm, Sweden; 2 Capio Spine Center Göteborg, Gothenburg, Sweden; 3 Pain in Motion Research Group (PAIN), Department of Physiotherapy, Human Physiology and Anatomy, Faculty of Physical Education & Physiotherapy, Vrije Universiteit Brussel, Brussels, 1050 Belgium; 4 Karolinska University Hospital, Theme Women’s Health and Allied Health Professionals, Medical Unit Allied Health Professionals, Solna, Sweden; 5 Department of Orthopaedics, Institute of Clinical Sciences at Sahlgrenska Academy, University of Gothenburg, Gothenburg, Sweden; 6 Department of Orthopaedics, Sahlgrenska University Hospital, Gothenburg, Sweden; 7 Institute of Health and Care Sciences, Sahlgrenska Academy, University of Gothenburg, Gothenburg, Sweden; 8 University of Gothenburg Centre for Person-Centred Care (GPCC), Sahlgrenska Academy, University of Gothenburg, Gothenburg, Sweden; 9 Region Västra Götaland, Research, Education, Development and Innovation, Primary Health Care, Gothenburg, Sweden; 10 Department of Clinical Neuroscience, Karolinska Institutet, Stockholm, Sweden; 11 Chronic Pain Rehabilitation, Department of Physical Medicine and Physiotherapy, University Hospital Brussels, 1050 Brussels, Belgium; 12 Department of Health and Rehabilitation, Unit of Physiotherapy, Institute of Neuroscience and Physiology, Sahlgrenska Academy, University of Gothenburg, Gothenburg, Sweden

**Keywords:** Telerehabilitation, Rehabilitation, Lumbar spine, Decompression surgery, Pilot, Treatment fidelity, Accelerometry, Steps per day

## Abstract

**Background:**

Lumbar spinal stenosis is characterized by walking limitations that often lead to physical inactivity and potentially associated health risks. This trial aimed to examine whether a person-centered digital program targeting physical activity (Get Back feasibility) was feasible and whether it contributed to clinically meaningful improvements in intervention outcomes among patients with LSS who were undergoing surgery.

**Methods:**

A two-arm randomized feasibility trial included physically inactive patients ≥ 18 years with central lumbar spinal stenosis scheduled for decompression surgery. The participants were randomized to Get Back or usual physical therapy. The 12-week intervention comprised person-centered support for behavioral changes in physical activity through video and telephone calls with a physical therapist. The feasibility outcomes included process feasibility, resource feasibility, and treatment fidelity, based on data from screening lists, study-specific questions, patient-reported outcome measures, and semi-structured interviews. The outcomes related to the intervention content included objectively assessed steps per day and physical activity, as well as self-reported fear of movement, pain catastrophizing, and general self-efficacy. Process and resource feasibility, as well as tentative changes in post-intervention outcomes, were assessed and reported using descriptive statistics. The temporal relationships of variables during the intervention were analyzed exploratively using cross-lagged correlations. Treatment fidelity, including treatment dose and adherence to the person-centered approach, was evaluated using descriptive statistics and a mixed-methods approach, respectively.

**Results:**

Of the 226 screened patients, 43% (*n* = 98) fulfilled the screening criteria. Of those, 67 were asked to participate, and 29 were randomized. The most common reason for declining participation was not wanting a digital intervention. The participants found the video format and outcome measures relevant and useful. The response rates were high (92–100%), except for the accelerometer follow-up (76%). The planned primary outcome for the future randomized controlled trial, steps per day, showed tentative between-group differences in favor of the intervention group. In both groups, fear of movement and pain catastrophizing decreased. The intervention participants attended four video sessions and a median of four telephone sessions (3–5). The physical therapists performed the intervention as planned, with fidelity to the person-centered approach, and behavior-change techniques were used.

**Conclusions:**

Get Back was feasible for patients with lumbar spinal stenosis who were receiving decompression surgery, with some modifications to strengthen the overall study procedure and intervention before proceeding to a full-scale randomized controlled trial.

**Trial registration:**

Registered at ClinicalTrials.gov, 04/08/2023, registration no. NCT05806593.

**Supplementary Information:**

The online version contains supplementary material available at 10.1186/s40814-026-01826-6.

## Key messages regarding feasibility

- *What uncertainties existed regarding the feasibility?* Whether a person-centered digital rehabilitation program would be feasible and acceptable for physically inactive patients undergoing lumbar spinal stenosis surgery. Specifically, regarding willingness to participate in a digital intervention, the optimal timing and dose of rehabilitation, appropriate outcome measures, and whether physical therapists could deliver a person-centered cognitive behavioral therapy intervention via video and telephone calls.
- *What are the key feasibility findings?* The findings concerning process feasibility, resource feasibility, and treatment fidelity support a full-scale trial with modifications. Exploratory analyses of steps per day informed the decision to retain this metric as the planned full-scale trial’s primary outcome.
- *What are the implications of the feasibility findings for the design of the main study?* Before a full-scale randomized controlled trial (RCT), modifications are needed, including revisiting the screening criteria, reducing the number of patient-reported outcome measures (PROMs), enhancing the accelerometer follow-up completion rate, incorporating digital support during recruitment, updating intervention sessions’ distribution, and clarifying the co-creation documentation for the person-centered approach**.**

## Background

Lumbar spinal stenosis (LSS) affects approximately 103 million people globally, with an annual incidence of 1.4% [1]. The condition predominantly occurs after 60 years of age. LSS is characterized by back and leg pain that is aggravated during walking [2], and it often leads to physical inactivity. If other efforts to reduce symptoms fail, surgery may be justified [3]. One year after LSS surgery, 23% of patients report unchanged or worse leg pain, 29% are unable to walk more than 100 m [4, 5], and 90% do not meet healthy physical activity recommendations [6]. These suboptimal outcomes underscore the need for well-designed rehabilitation studies [7] investigating whether rehabilitation combined with surgery can improve outcomes.

Promoting healthy physical activity is a key target for improving recovery and the outcomes of spine surgery [8]. Since walking limitations are LSS’s cardinal symptoms, and physical inactivity is linked to adverse health outcomes, rehabilitation should prioritize restoring and encouraging walking behavior. This approach aligns with the physical activity guidelines of the World Health Organization (WHO), which recommend that adults engage in at least 150 min of moderate-intensity physical activity, or 75–150 min of vigorous-intensity per week [9]. Physical activity levels and health benefits share a clear dose-response relationship, with the least physically active people benefitting the most [10]. Identifying and supporting physically inactive patients is, therefore, essential for optimal rehabilitation and lasting well-being.

Step count is a common and accessible method for assessing physical activity [11]. A recent meta-analysis that included a general population concluded that a 1000-step increase per day is associated with an approximately 15% reduction in all-cause mortality and that a 500-step incremental increase is associated with approximately 7% decreased cardiovascular mortality [12]. This finding highlights physical activity’s general importance for all people, regardless of health condition. Modifiable psychological factors, including pain catastrophizing and fear-avoidance beliefs, are associated with lower daily step counts among patients with LSS [13]. Among patients with chronic low back pain and degenerative disc disorder, fear of movement is associated with postsurgical sedentary behavior [14].

Accordingly, we designed Get Back as a person-centered program to support inactive patients in becoming physically active after decompression surgery [15]. Get Back builds on the framework and findings from a previous RCT [16–18]. In that RCT, we identified distance to the rehabilitation clinic as a common reason for declining participation. The Get Back intervention is delivered digitally to facilitate equality and access to postoperative spine rehabilitation.

This study aimed to examine, before a full-scale RCT, whether the current format, Get Back feasibility, is feasible in terms of process and resources, whether it contributes to clinically meaningful improvements in intervention-content-related outcomes, and whether it ensures treatment fidelity for patients undergoing LSS surgery. Table 1 presents the research questions.

**Table 1:** Research questions previously published in the study protocol, Karlsson et al. [15]

Research questions related to:	
1. Process and resource feasibility	1.1 What percentage of patients planned for decompression surgery for lumbar spinal stenosis meeting inclusion criteria are eligible after the screening procedure? 1.2 What are the reasons for declining participation in the study or dropping out? 1.3 Is the screening questionnaire measuring physical activity level able to detect patients planned for decompression surgery for lumbar spinal stenosis with a low level of physical activity compared to accelerometer data at baseline? 1.4 How many of the planned sessions of the Get Back intervention do patients of the intervention group attend? 1.5 Did the study participants and physical therapists in the study find the digital format, Get Back intervention, and outcome measures relevant and usable? 1.6 Is the Get Back treatment safe (type and frequency of adverse events) in patients undergoing decompression surgery for lumbar spinal stenosis? 1.7 What is the response rate of the patient reported outcome measures (PROMs) and to what extent are physical tests completed in patients undergoing decompression surgery for lumbar spinal stenosis? If they are not completed, what are the reasons?
2. Outcomes related to the Get Back content	2.1 Do the assessments preoperatively and at 12-week follow-up of steps per day, physical activity and pain catastrophizing, fear of movement and general self-efficacy provide tentative information as to the efficacy of the Get Back intervention in patients undergoing decompression surgery for lumbar spinal stenosis? 2.2 Do the weekly assessments of single-item questions aiming to measure steps per day, physical activity and aspects of pain catastrophizing, fear of movement and self-efficacy provide additional information regarding the efficacy of the Get Back intervention, trajectories of change and interrelations between variables in patients undergoing decompression surgery for lumbar spinal stenosis?
3. Treatment fidelity of the Get Back intervention	3.1 Is the treatment dose and content of Get Back delivered as intended in patients undergoing decompression surgery for lumbar spinal stenosis? 3.2 Does the physical therapist delivering the Get Back intervention adhere to a person-centered approach?

## Methods

### Study design

A feasibility trial with a two-arm randomized design (1:1 allocation) was conducted. The study was reported according to the Consolidated Standards of Reporting Trials (CONSORT) extension for randomized pilot and feasibility trials [19] (see Additional file 2 for a checklist).

### Participants, setting, and recruitment

Patients ≥ 18 years of age with central LSS who were scheduled for decompression surgery (without concomitant fusion surgery) were considered for inclusion. Patients with malignancy, severe neurological or rheumatic disease, idiopathic scoliosis, isthmic spondylolisthesis, the inability to understand written information or communication in Swedish, or untreated or unstable heart disease that hindered physical capacity tests were excluded.

Eligible patients were initially screened for physical inactivity (using two self-report questions from the National Board of Health and Welfare in Sweden, which were converted into the WHO’s health-promoting recommendations of > 150 min/week of moderate physical activity [20]), and high levels of fear of movement (≥ 37 on the Tampa Scale of Kinesiophobia, TSK) or pain catastrophizing (≥ 30 on the Pain Catastrophizing Scale, PCS). Thus, this study defined *physical inactivity* as not achieving the > 150 min/week recommendation for moderate physical activity. After 3 months of recruitment, only six out of 50 patients were identified as eligible. The screening criteria were revised in mid-August 2023 to screen for physical inactivity only. The “Discussion” section elaborates further. Between April 17, 2023, and June 30, 2024, 29 participants were consecutively recruited from two spine clinics with referrals from different Swedish regions. Eligible participants were given oral and written study information, and they provided written informed consent upon agreeing to participate.

### Procedure

After inclusion, a preoperative baseline assessment was conducted. It included a seven-day accelerometry registration for physical activity levels, three physical-capacity tests, and PROMs. The study coordinator distributed the accelerometer by mail. Two independent observers (physical therapists) conducted a video assessment (the Doctrin platform, Doctrin AB, Sweden), collecting demographic/clinical data and three physical-capacity tests. The PROMs were answered using the BASS platform from the eHealth Core Facility at Karolinska Institutet, Sweden. After baseline assessments, the participants were randomly assigned to either the intervention group with Get Back or the control group with the usual physical therapy, as described under the heading Study arms. The assessments were repeated at follow-up 11–12 weeks postoperatively. The procedure is illustrated in our study protocol [15].

Between the baseline and the 11–12-week follow-up, the participants answered nine single-item questions in the BASS platform weekly regarding steps per day, fear of movement, pain catastrophizing, and self-efficacy concerning physical activity. After data collection, semi-structured telephone interviews with the intervention participants explored their experience with the intervention and evaluated the data collection strategies’ feasibility. The control group and independent observers completed a corresponding questionnaire about the data collection strategies.

### Study arms

Get Back was conducted through five video meetings and five telephone calls over 12 weeks, from 1 week preoperatively until 11 weeks postoperatively. The intervention was guided by two trained study physical therapists experienced in evidence-based pain rehabilitation. They followed a core structure in a study-specific manual. Get Back aimed to increase steps per day (a proxy for physical activity), built on a person-centered approach, behavior-change techniques (BCTs), and fear-avoidance factors. A pedometer (OMRON HJ-322U WS Pro 2.0) was used for steps-per-day self-monitoring. This intervention is described in detail elsewhere [15]. Control-group participants received the usual physical therapy, according to the regimen at each participating spine clinic, which commonly includes preoperative information and referrals to outpatient physical therapy. During postsurgical hospitalization, both groups met a physical therapist once in the ward and received similar care at both recruiting sites. This meeting included advice on gradually increasing activity and handling various types of postoperative pain, as well as instructions for exercises to activate core and leg muscles and perform functions of daily living, such as rising from sitting.

### Process and resource feasibility

Recruitment aspects were reported as the percentage of eligible patients after the screening procedure and reasons for declining or dropping out of the study. To investigate whether the screening questions could detect physically inactive patients, the questions were compared with the objective accelerometer data at the baseline. Intervention use was reported as the number of completed versus scheduled treatment sessions. The participants and physical therapists jointly agreed upon scheduling sessions, according to the participants’ needs. The digital format’s usability was evaluated via a specific question from the semi-structured interviews. The data collection methods’ usability was evaluated through study-specific questions for the participants and independent observers. These questions were formulated through clinical reasoning to cover all assessments’ relevance and usability. The data collection methods evaluated for this were as follows.

- Physical activity: accelerometry [21].
- Physical capacity tests: one-leg stand [22], timed up-and-go test [23], and 30 s sit-to-stand test [24].
- PROMs: the person’s own activity goals rated using the Patient-Specific Functional Scale [25]; health-related quality of life using the EQ-5D-3L, including the EQ VAS [26]; general self-efficacy using the General Self-Efficacy Scale (GSE) [27]; fear of movement using the TSK [28]; pain catastrophizing using the PCS [29]; back-related disability using the Oswestry Disability Index (ODI) [30]; and depressive mood using the depression subscale of the Hospital Anxiety and Depression Scale [31].

The physical therapists reported on treatment safety (possible adverse events during the intervention) after each session.

### Outcomes related to the Get Back content

In this feasibility study, post-intervention outcomes included the planned primary and secondary outcomes intended for the future RCT. Planned primary outcome measures: a triaxial waist-worn accelerometer (ActiGraph GT3X +; ActiGraph, Pensacola, FL, USA) was used to assess physical activity. We used steps per day and moderate-to-vigorous physical activity (MVPA). The participants were instructed to wear the accelerometer when awake for seven consecutive days [21]. The clinically meaningful number of steps was an improvement of 1000 steps/day [12]. Planned secondary outcome measures: Pain-related catastrophizing was reported using the 13-item PCS, with a total score of 0–52 and a higher score indicating a higher degree of catastrophizing thoughts [29]. Fear of movement was measured using the 17-item TSK, yielding a sum between 17 and 68; a higher value indicated a higher degree of fear of movement [28]. General self-efficacy was measured using the 10-item GSE, generating a score of 10–40; a higher score represents better general self-efficacy [27]. The planned primary and secondary outcomes were assessed at baseline and 11–12 weeks postoperatively. Through nine single-item questions, the participants self-reported once a week on the number of steps per day based on pedometer data (outcome variable), numeric rating scale (NRS) for pain, PCS items 8 and 5, TSK items 7 and 12, and self-efficacy related to pain, activity, and falls (process variables).

### Treatment fidelity

The physical therapists reported on each session’s treatment components using a checklist based on the treatment manual. The checklist included BCTs and the three routines from the Gothenburg framework (initiating, working, and safeguarding partnership) for a PCC approach [32]. To further evaluate physical therapists’ adherence to this approach, participant data from a single question and text data from semi-structured interviews on participants’ perceptions of the PCC approach were collected. To strengthen fidelity, two workshops based on case reports and audio recordings were held with interventionists and two advisers experienced in PCC and cognitive behavioral therapy. The treatment dose was assessed based on the number of sessions attended and each session’s length in minutes, per the physical therapists’ report.

### Sample size

To evaluate feasibility, as stated in the research questions of this two-arm trial [33], 24 participants were determined to be sufficient [34]. To allow for withdrawals after inclusion, we estimated 15 participants per group (*n* = 30 in total).

### Randomization procedure

An independent statistician not involved in outcome assessment generated a computer-randomized 1:1 allocation sequence stratified by clinic using SAS PROC PLAN (simple randomization plan). Allocation concealment was maintained via a computerized randomization list accessible only to authorized study personnel after enrollment. We prepared opaque, sequentially numbered, sealed envelopes off-site. The envelopes were stored in a locked cabinet and opened sequentially after enrollment by a study coordinator not involved in assessments or the intervention. When baseline measurements were completed, the study coordinator randomized the participants. Participants’ or interventionists’ blinding was not possible because of the intervention’s nature. The orthopedic surgeons and the assessing physical therapists were blinded.

### Analyses

Demographic and clinical data were reported as counts, proportions, percentages, medians with interquartile ranges (IQR), and means with standard deviations (SD) as appropriate.

Process and resource feasibility were analyzed descriptively; rates regarding eligibility, recruitment, and screening were reported as percentages with corresponding counts, whereas rates regarding response and completion were reported with proportions and percentages. Intervention use, including the number of completed core and booster sessions, was summarized using medians and IQR. Adverse events were reported as counts. Interview data regarding the usability of the intervention’s digital format were categorized through coding in a descriptive content analysis manner [35].

To address the outcomes related to the intervention content, tentative changes in physical activity and psychological variables from the baseline to the follow-up were reported descriptively for the total study sample and each group: accelerometer data were analyzed as described by Karlsson et al. [15]. In accordance with recommendations for pilot and feasibility studies, no statistical tests were conducted for between-group efficacy. Analyses to quantify uncertainty of clinical outcomes were conducted in R, version 4.5.1 (R Core Team, 2024). For normally distributed data, we summarized central tendency using the mean (SD) and calculated 95% confidence intervals (CIs) using the *t*-distribution (mean ± *t*·SE). For skewed distributions, we summarized central tendency using the median (IQR) and calculated the 95% CIs using exact, distribution-free, binomial-based intervals for ordered observations. For change scores, we first calculated the within-subject difference (post-pre) for each participant. Then, we summarized these individual differences, rather than using the difference between group-level pre-operative and post-operative summaries, to more accurately reflect paired data—particularly in small and heterogeneous samples [36]. Repeated weekly measures were analyzed visually through trend, level, and variability assessments, and they were graphed at the group level using median scores. Trend lines were added to aid in the visual analysis of the change trajectories during the intervention. Cross-lagged correlation analyses were utilized to explore interrelations between each process variable and the outcome variable, steps per day, using Simulation Modeling Analysis (SMA, Version 7.3.2, https://www.clinicalresearcher.org/) [37]. The SMA is a nonparametric method based on bootstrapping that adjusts for autocorrelation in time-series data. Temporal correlations were explored from *lag* − 2 to + 2; a zero lag indicates a simultaneous change in both variables, positive lags indicate that process variables precede changes in steps per day, and negative lags indicate that process variables follow such changes.

Treatment dose and use were analyzed through the frequencies of delivered content (e.g., the use of health plans and BCTs), the median number of sessions, and the sessions’ mean (SD) duration. A convergent mixed-methods approach for feasibility studies [38] was employed for research question 3.2 (“Does the physical therapist delivering the Get Back intervention adhere to a person-centered approach?”) for a more comprehensive understanding (completeness) [38]. Specifically, a convergent parallel mixed-methods design was used [39], in which quantitative and qualitative data were collected concurrently, analyzed separately, and merged at the interpretation stage. Quantitative data were analyzed descriptively with proportions and medians, as appropriate. Qualitative data from interviews (*n* = 12, 17–33 min), transcribed verbatim by E.K., were analyzed using deductive content analysis, according to the description by Elo and Kyngäs [35]. M.L. and C.E. selected the units of analysis and made sense of the data. A structured categorization matrix was created with categories based on the Gothenburg framework’s three routines (initiating, working, and safeguarding the partnership) for the PCC approach [32]. The selected data were coded according to the categories M.L. and C.E. assigned, and C.E. grouped and subcategorized the codes, which M.L. and E.K. verified. The qualitative and quantitative results were integrated through merging and reported using a joint display [38].

## Results

### Process and resource feasibility

**Fig. 1:**
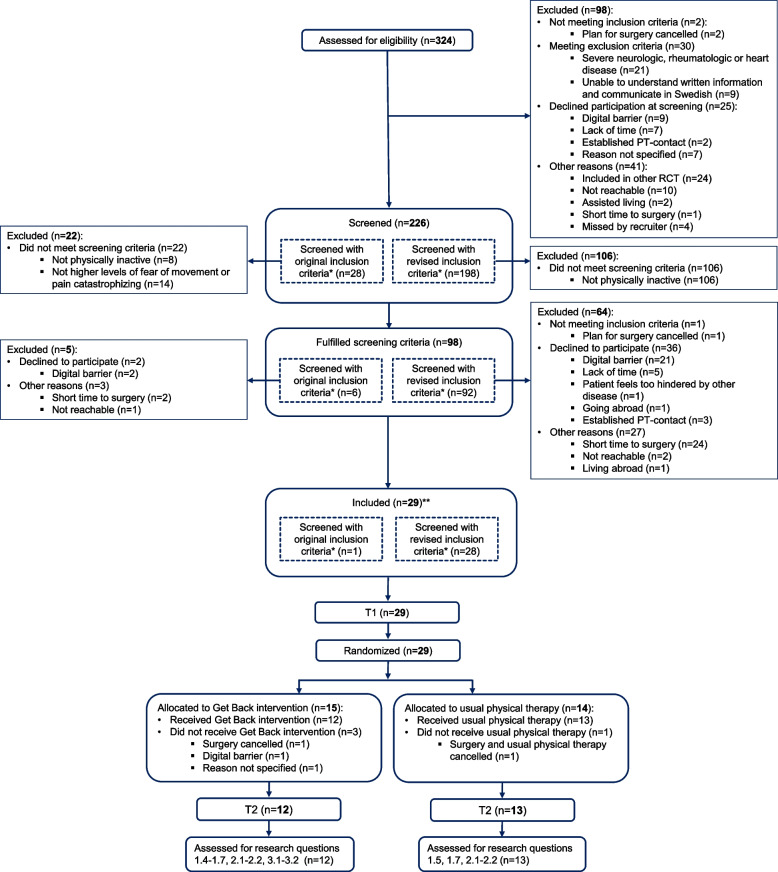
CONSORT flow diagram. * = original screening criteria; < 150 min/week of moderate physical activity, and ≥ 37 on the Tampa Scale of Kinesiophobia or ≥ 30 on the Pain Catastrophizing Scale. Revised screening criteria; < 150 min/week of moderate physical activity. ** = 1 participant was included on the original screening criteria. Abbreviations: PT = physical therapist, TSK = Tampa Scale of Kinesiophobia, PCS = Pain Catastrophizing Scale, T1 = Baseline assessment, T2 = follow-up assessment

After the screening procedure, 43% (*n* = 98) of patients fulfilled the screening criteria. Of 67 patients who were asked to participate, 43% (*n* = 29) were included in the study (Fig. 1). The 38 patients who declined participation had a mean age of 74.7 years (SD 8.5), and 21 (55%) were women. The most frequent reason for this decline was not wanting to use a digital format (*n* = 9 before screening and *n* = 24 after screening). The median time from the baseline assessments to surgery was 14 days (IQR 8–20). After randomization, two surgeries were canceled, and two participants withdrew (Fig. 1).

The intervention-group participants have a higher mean age (71 years) than the control group (64 years; Table 2). Control-group participants (*n* = 13) reported seeing a physical therapist a median of three times (0–8) during the 12-week period, whereas three did not see a physical therapist at all. One intervention-group participant experienced postoperative complications (wound infection and re-operation) within 3 months. All intervention participants attended four core sessions and a median of four booster sessions (3–5); for further details see treatment dose under treatment fidelity. No adverse events were reported during the intervention.

**Table 2:** Participants’ (*n* = 25) demographic and clinical characteristics

Variable	Intervention group (n = 12)	Control group (n = 13)	Total (n = 25)
Age, years, mean (SD)	71 (7)	64 (10)	68 (9)
Gender, *n *(%)			
Female	8 (67)	9 (69)	17 (68)
Male	4 (33)	4 (31)	8 (32)
Non-binary	0 (0)	0 (0)	0 (0)
Living situation, *n* (%)			
Living alone	6 (50)	3 (23)	9 (36)
Living with partner	6 (50)	10 (77)	16 (64)
Lifestyle factors			
Smoking, *n* (%)			
Previously	2 (17)	8 (61.5)	10 (40)
No	10 (83)	5 (38.5)	15 (60)
Alcohol use, *n* (%)			
≥ 2 units/day	0 (0)	1 (8)	1 (4)
< 2 units/day (yes/no)	12 (100)	12 (92)	24 (96)
BMI, mean (SD)	28.0 (5.1)	29.6 (3.9)	28.8 (4.4)
Previous spine surgery, *n* (%)			
Yes	3 (25)	2 (15)	5 (20)
No	9 (75)	11 (85)	20 (80)
Occupation, *n* (%)			
Employed	2 (17)	5 (38.5)	7 (28)
Retired	10 (83)	8 (61.5)	18 (72)
Analgesic use, *n* (%)			
Yes	9 (75)	11 (85)	20 (80)
No	3 (25)	2 (15)	5 (20)
Leg pain, *n *(%)			
Yes	11 (92)	13 (100)	24 (96)
No	1 (8)	0 (0)	1 (4)
Back pain, *n* (%)			
Yes	10 (83)	10 (77)	20 (80)
No	2 (17)	3 (23)	5 (20)
Pain duration, years, median (IQR), *n* = 18 (9 per group) due to missing values			
Leg	1.0 (1.0–2.0)	2.0 (1.0–2.8)	1.25 (1.0–2.5)
Back	2.5 (0.8–6.0)	2.0 (1.3–6.0)	2.25 (1.0–5.5)
Pain intensity, NRS, median (IQR)			
Leg	7 (6–8)	8 (6–8)	7 (6–8)
Back	8 (6–8)	7 (4–8)	7 (5–8)
Education, *n* (%)			
Compulsory level	1 (8.3)	1 (8)	2 (8)
High school graduation	1 (8.3)	6 (46)	7 (28)
Vocational education	1 (8.3)	2 (15)	3 (12)
University	9 (75)	4 (31)	13 (52)
Comorbidity CCI, median (IQR)	0 (0–1)	1 (0–3)	1 (0–2)
HRQoL, EQ5D-VAS, mean (SD)	55 (21)	53 (21)	54 (21)
HADS-D, median (IQR)	2 (1–8)	4 (1–10)	3 (1–9)
ASA score, *n* (%)			
I	3 (25)	1 (8)	4 (16)
II	6 (50)	8 (61)	14 (56)
III	3 (25)	4 (31)	7 (28)

*Abbreviations*: *IQR* Interquartile range, *NRS* Numerical Rating Scale (0–10), *CCI* Charlson Comorbidity Index, *EQ5D-VAS* EuroQol version 5-dimension visual analogue scale (0–100, with 100 = Best imaginable health state), *HADS-D* the depression subdomain of the Hospital Anxiety and depression scale (0–21, cut-off 8 for depressive mood), *ASA* American Society of Anesthesiologists

Three out of 25 participants (12%) who self-reported physical inactivity at screening attained MVPA ≥ 150 min/week, thus achieving health-promoting physical activity when evaluated with the accelerometer.

At the baseline, the PROMs response rate was *n* = 29/29 (100%), the completion rate of the video assessments was *n* = 25/25 (100%), and that of the accelerometer assessments was *n* = 23/25 (92%). One participant was sick during the assessment, and one accelerometer was lost in the mail. The weekly questionnaire’s response rate was 282 of 285 (99%). One participant missed three questionnaires due to postoperative complications. The response and completion rates of the follow-up PROMs and video assessments were *n* = 25/25 (100%). The follow-up accelerometer assessment was completed by *n* = 19/25 (76%) participants; two did not wear the accelerometer, two were staying temporarily at other addresses, and two provided only two valid days of data.

**Fig. 2:**
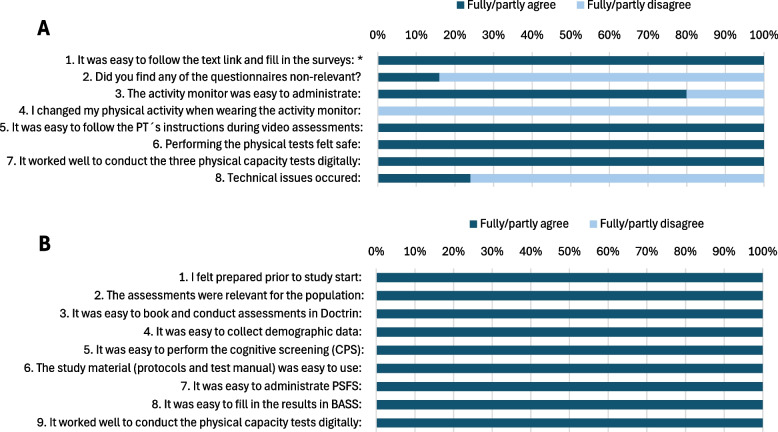
**a** Participants’ usability-outcome measures (*n* = 25), *with two not answering this question. **b** Independent observers’ usability-outcome measures (*n* = 2)

Overall, the participants found the PROMs and digital physical capacity tests relevant and usable (Fig. 2a). The challenges they reported included other pain conditions that influenced PROM responses, too many PROMs, and minor technical and activity monitor issues. The independent observers found the data collection methods relevant and usable overall (Fig. 2b).

Half the intervention group explained that the video format enabled visual contact and feedback that strengthened the rehabilitation process (comparable to the telephone, and equal to or better than physical meetings). The digital format’s most-mentioned benefit was “not having to leave home or travel,” which 10 of 12 interviewees mentioned. Participants stated that the remote location enhanced accessibility, provided a sense of freedom and participation, enabled rehabilitation in daily activities, facilitated the establishment of routines, and offered rehabilitation in a safe environment with fewer “disturbing” factors. One participant described preferring physical contact and perceived people as becoming distant because of technology. Four of 12 participants felt that the digital format’s suitability required certain prerequisites, such as self-discipline, experience with rehabilitation exercises, and technical knowledge.

### Outcomes related to the Get Back content

Both groups showed improvements in all intervention-content-related variables from the preoperative baseline to 11–12 weeks postoperatively (Table 3). The absolute median improvements in steps per day and MVPA were greater in the intervention group than in the control group. The absolute median improvement in general self-efficacy was higher in the control group (Table 3).

**Table 3:** Intervention-content-related variables at the baseline and 11–12 weeks postoperatively in the intervention and control groups

		Intervention group				Control group		
	*n*	Preop (baseline)	11–12 weeks postop	Absolute* change	*n*	Preop (baseline)	11–12 weeks Postop	Absolute^*^ change
**Planned primary outcome measures**								
**Steps per day**								
Median (IQR) 95% CI (LB, UB)	*11*	3198 (2579–4007) (2069, 4276)	4401 (3519–6090) (3439, 6319)	1496 (− 814–2821) (− 837, 3158)	*7*	3676 (2559–7929) (2473, 9533)	5017 (2144–7180) (1612, 8407)	124 (− 2064–1173) (− 3114, 4707)
**MVPA min/day**								
Median (IQR) 95% CI (LB, UB)	*11*	1.6 (0.7–4.6) (0.6, 6)	4.0 (1.5–7.6) (0.6, 20.7)	2.4 (− 0.3–7.0) (− 2, 20)	*7*	2.9 (1.4–22.8) (0.4, 24.4)	10.2 (0.7–27.7) (0.3, 32.1)	0.3 (− 1.2–7.7) (− 7, 25)
**Planned secondary outcome measures**								
**PCS**								
Mean (SD) 95% CI (LB, UB)	*12*	16 (10) (9, 22)	7 (5) (4, 10)	− 9 (8) (− 14, − 4)	*13*	19 (10) (13, 25)	9 (8) (4, 14)	− 9 (9) (− 15, − 4)
**TSK-SV**								
Mean (SD) 95% CI (LB, UB)	*12*	35 (6) (31, 39)	27 (5) (23, 30)	− 8 (6) (− 12, − 4)	*13*	36 (8) (32, 41)	28 (7) (24, 32)	− 8 (4) (− 11, − 6)
**GSE**								
Mean (SD) 95% CI (LB, UB)	*12*	32 (3) (30, 34)	34 (4) (31, 36)	1 (6) (− 3, 5)	*13*	31 (5) (28, 34)	35 (3) (33, 37)	4 (6) (0, 7)

*Abbreviations*: *IQR* Interquartile range, *95% CI (LB, UB) *95% confidence interval (lower bound, upper bound), *MVPA* Minutes in moderate-vigorous physical activity, *PCS* Pain Catastrophizing Scale (0–52, lower = better), *TSK-SV* Tampa Scale of Kinesiophobia Swedish (17–68, lower = better), *GSE* General Self-Efficacy Scale (10–40, higher = better)

^*^Change scores are based on individual medians or means and not the group medians or means reported for pre- and postoperative values

The relative change in steps per day (based on each participant’s proportion of change) was, on average, 50% in the intervention group and 17% in the control group. Five of 11 intervention-group participants (45%) increased their daily steps by ≥ 1000, versus 2 of 7 (29%) in the control group. In the intervention group, 7 of 12 (58%) participants reached the MCID/MIC (a decrease of > 6) in the TSK [40] and (≥ 8) in the PCS [41], versus 8 of 13 (62%) for the control group.

**Fig. 3:**
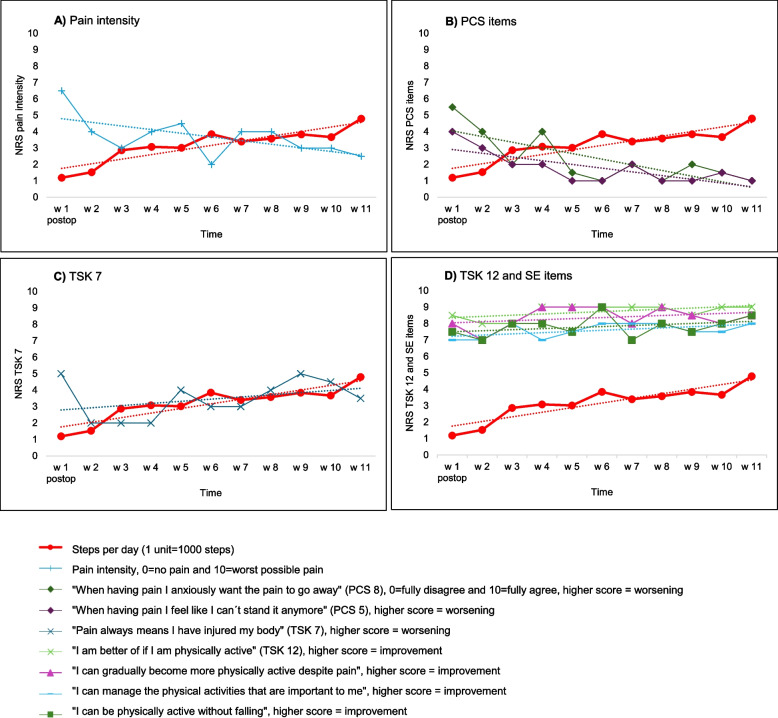
Trajectories of the process variables and the planned outcome (steps per day), including trendlines. Reported as the weekly median of each variable in the intervention group (*n* = 11–12). All variables were rated on a numeric 0–10 scale, where 0 = fully disagree and 10 = fully agree, except for steps per day. **a** Pain intensity and steps per day. **b** Pain catastrophizing items and steps per day. **c** Tampa Scale of Kinesiophobia question 7 and steps per day. **d** Tampa Scale of Kinesiophobia question 12, pain self-efficacy, self-efficacy for activity, self-efficacy for falls, and steps per day

Steps per day increased postoperatively for most participants throughout the intervention (Fig. 3a–d). At the group level, the following pattern was noted between the process variables and the planned outcome, variable steps per day, during the intervention’s postoperative period: Pain severity decreased (negative trend, Fig. 3a) simultaneously with the increase in steps per day and items PCS-5 and PCS-8 (negative trends, Fig. 3b). Four single items, the TSK-12, pain self-efficacy, physical activity self-efficacy, and self-efficacy for falls, showed limited improvements from already high ratings (weak positive trends, Fig. 3d). The TSK-7 showed a variable pattern during the intervention, with no improvement (positive trend, Fig. 3c). The Spearman’s rho correlation coefficients, the highest of the assessed time lags, for steps per day were − 0.78 for NRS (*lag 0*), − 0.77 for PCS Q8 (*lag 0*), − 0.79 for PCS Q5 (*lag 0*), 0.67 for TSK Q7 (*lag − 1*), 0.58 for TSK Q12 (*lag* + *1*), 0.45 for SE pain (*lag* + *2*), 0.56 for SE PA (*lag 0*), and 0.61 for SE fall (*lag 0*). A full report of all assessed time lags is available in Additional file 1. The group-level summarized results do not apply to each participant, and patterns varied considerably among the participants. Overall, 11 of 12 participants showed improvements or no changes in process measures, and one participant showed a decline.

### Treatment fidelity

All intervention participants attended four core sessions and initiated and documented a health plan. For most, the second core session before surgery was omitted (*n* = 8) or replaced with a short phone call (*n* = 4) due to patient preferences and a short time before surgery. Person-centered communication about pain was conducted throughout the intervention, based on each patient’s needs and current symptoms. On average, the first core session lasted 47 min, and the following sessions lasted 32 min. The median number of booster sessions was four (3–5), with an average duration of 22 min. One participant rescheduled three sessions due to postoperative complications. All participants used a pedometer to self-monitor steps per day and a physical activity diary. Table 4 presents the BCT [42] use during the sessions.

**Table 4:** Behavior-change techniques in session checklists for *n* = 12 intervention-group participants

	AP	SM	RG	GT	FB	IH	BP	BE	EXP
Core 1 (%)^a^	100	100	NA	0	33	100	0	0	0
Core 3–5 (%)^b^	100	100	100	92	100	92	11	19	8
Boosters (%)^c^	100	98	93	87	98	59	2	11	11

*Abbreviations*: *AP* action planning (no. of behavior change technique component in the taxonomy by Michie et al. 2013: 1.4), *SM* self-monitoring (2.3), *RG* review of behavior goals (1.5), *GT* graded tasks (8.7), *FB* feedback on behavior (2.2), *IH* information about health consequences (5.5), *BP* behavioral rehearsal/practice (8.1), *BE* behavioral experiment (4.4), *EXP *exposure (7.7)

^a^* n* = 12 sessions in total

^b^*n* = 36 sessions in total

^c^*n* = 46 sessions in total

The physical therapists seem to have followed a person-centered approach during the intervention, with strong indications of mutual partnership and active participation between the participant and the physical therapist (Table 5). However, in safeguarding this partnership, the participants also described other methods than the health plan to document adaptation and physical activity progress.

**Table 5:** Joint display of quantitative and qualitative findings to describe the physical therapists’ adherence to the person-centered approach

	Quantitative	Qualitative		Meta-inferences
The Gothenburg framework’s three routines for a person-centered care approach [32]		**Subcategories** The participants experienced a person-centered approach…	**Illustrative quotes from interviews**	**Interpretation of mixed-methods findings**
Initiating the partnership: “This involves listening carefully to the patient’s story to understand their condition, their capabilities, and resources as well as obstacles to achieving good health, giving due consideration to diagnoses and treatments.”	Study PTs reported 100% adherence to initiating the partnership	…as the PT actively listened to the participants’ stories … as establishing a personal connection was important for continued rehabilitation	“The PT has been responsive to the things that I told, how I have felt in different situations.” “We got to meet once before [the operation] and got to know each other, and the PT got to know how I felt at that time and could then follow my improvements.”	The study PTs reported high adherence to all three routines, suggesting a person-centered approach in the partnership. Those findings were complemented by the participants’ experiences in initiating, working, and safeguarding the partnership and high self-reported perceived participation, except for aspects concerning co-creating and documenting adaptations in the health plan. More often, participants mentioned documentation and adaptation concerning the activity diary, oral discussions, and goal formulations. This suggests a need for clarification regarding the terminology and use of the health plan
Working the partnership: “to co-create a personal health plan consistent with identified resources and barriers and combined with medical and health research evidence. This partnership is intended to support the patient’s self-efficacy and self-management by paying attention to their own priorities and building on their capabilities.”	Study PTs reported 100% adherence to working the partnership and co-creating a health plan with all participants at Core Session 1 (*n* = 12/12) Intervention participants reported: 100%) reported yes for perceived self-participation^*^ throughout the intervention period (*n* = 12/12)	…by expressing the value of attending sessions regularly and taking part in planning the schedules …by feeling communion with and trust in the PT …as individual resources for and barriers to physical activity were considered	“It is very good to have someone who is regularly in contact with you, who follows you. It is this continuity, that you know that, in 2 weeks, I can ask this when we have another conversation.” “It was good to get feedback from the PT. I could express my thoughts and where I was in the recovery process. I don't have anyone else at home either…and you can’t call your friends and complain that your back hurts all the time, so I think it was good that I was heard and understood about the struggles that I experienced with back pain and surgery.” “I have gotten so much valuable advice, which the PT has followed up on. And the next time asked, ‘How did it go in the forest? Have you taken such a walk? What problems did you encounter there? Or did it go well? Is it possible to continue? Can you do even longer distances?’ I have received very good feedback.”	
Safeguarding the partnership—documentation: “documenting the health plan, adapting it to changes in the patient’s goals and/or other circumstances over time and in different settings.”	Study PTs reported 100% adherence to safeguarding the partnership	…as they documented their physical activity progress in one way or another	“I’ve written every night what I’ve done…when I’ve done something special, I’ve written little comments about it in this diary.” “A health plan? I don’t remember that. What was in it?”	

*Abbreviations*: *PT* physical therapist

^*^= “Have you felt involved in your pre- and postoperative care/treatment?”

## Discussion

This study examined whether the Get Back program was feasible for patients undergoing decompression surgery due to central LSS. The findings on process feasibility, resource feasibility, and treatment fidelity support a full-scale trial with protocol refinements regarding recruitment, PROM selection, accelerometer follow-up, digital support, session distribution, and ensuring a person-centered approach. Exploratory analyses of steps per day informed the decision to retain this metric as the planned RCT’s primary outcome. No adverse events were directly attributed to the intervention, supporting the safety of progressing to a full-scale RCT. See Additional file 3 for progression assessment by domain.

### Process and resource feasibility

The initial eligibility rate raised concerns regarding the generalizability and clinical utility of an intervention reaching only 12% of the addressed population. Revising the criteria to include patients who were physically inactive increased the eligibility rate to 43%, aligning with the intervention’s primary aim. One potential reason for this eligibility rate was our physical-activity screening. At screening, 50% of patients reported fulfilling the WHO’s physical activity recommendations—higher than the previous 13% in a population of patients with LSS [43]. Thus, the screening method may not have captured all physically inactive patients efficiently. The two questions to self-report physical activity used in this study have previously shown a high degree of misclassification for patients after myocardial infarction [44]. Our study sample included a higher proportion of women compared to national data reported in the Swedish Spine Registry (68% vs. 52%) [5]; one potential reason could be our screening, as women tend to report being less physically active than men [45]. Excluding patients who were unable to understand written information or communicate in Swedish might have hindered health equity and introduced selection bias. This criterion was due to logistical constraints: soliciting written consent across languages was not manageable, all PROMs were not translated and culturally adapted, and resources could not obtain translators for the intervention sessions.

The digital format may have excluded older patients, introducing a selection bias, as those who declined participation were older than the participants. In a full-scale RCT, strategies such as digital coaches for technical support could improve inclusivity. A strength of the digital format is its ability to overcome geographical barriers to rehabilitation [16, 46]. Furthermore, the participants and independent observers found the format and outcome measures relevant and usable, aligning with previous research that highlighted the growing acceptance of home-based digital rehabilitation [47], specifically for spine rehabilitation [48].

Only two independent observers were employed, and while both found the outcome measures relevant and usable, their views might not be representative. Participant-reported challenges, such as too many PROMs, can be addressed by revisiting each PROM’s relevance and using shorter validated versions. Our response rates for PROMs were higher than those of previous studies in surgical settings [49]. A non-surgical study included older adults with musculoskeletal pain in a walking intervention with similar assessment procedures as ours, anticipating a 70% accelerometry follow-up rate and obtaining 67% valid accelerometer data at 12 weeks’ follow-up, versus our 76% [50]. Our strategies to enhance the completion rate at follow-up will include clarifying written accelerometer instructions, sending automatic reminders, and verifying addresses when mailing accelerometers.

A limitation of this study is the use of sealed opaque envelopes for allocation concealment, as envelope-based approaches are more susceptible to manipulation if not rigorously implemented [51]. To minimize this risk, envelopes were prepared off-site, stored securely, and opened sequentially only after enrollment [51].

### Outcomes related to the Get Back content

Following surgery, both groups showed overall improvements across the planned outcome measures—possibly reflecting natural postoperative recovery after LSS surgery. The intervention group showed larger improvements in daily steps, with higher relative and absolute changes and a greater proportion achieving a ≥ 1000 steps/day increase. These exploratory findings tentatively signal that the intervention may have helped increase daily steps, supporting the planned RCT’s use of steps per day as its primary outcome. However, as this was a feasibility study, not designed to calculate efficacy and including unbalanced groups, this interpretation merits caution. Between-group differences in age distribution and lifestyle factors, such as body mass index (BMI), may have influenced the observed group differences, as such factors significantly influence walking performance among patients with LSS [52]. Adequate randomization in a sufficiently powered RCT should mitigate these biases.

In our study, both groups demonstrated similar decreases in pain catastrophizing (PCS) and fear of movement (TSK); the findings should, therefore, be interpreted in the context of lower baseline levels and a potentially limited scope for change in this sample. The intervention seemed not to influence general self-efficacy. In the planned RCT, more context-specific measures—such as self-efficacy in relation to exercise [53] or pain [54]—may be more relevant, being more directly linked to physical activity behaviors [55].

The variability in weekly process measures highlights the need for a person-centered approach. Averaged across participants, daily steps rose rapidly in 1–3 weeks, plateaued in 4–7 weeks, then increased again in 8–11 weeks, while psychological items stabilized by week 4 and remained unchanged. Relocating a core session to the 4–7-week plateau phase may help precipitate earlier improvements. The continued increase in steps per day 11–12 weeks postoperatively suggests that the primary endpoint may need to be postponed to fully capture intervention efficacy. However, this trend is based on self-reported steps per day and should be interpreted cautiously. In addition, other studies on behavior-change interventions frequently adopt 12-week protocols [56]. TSK Item 7 (“Pain always means I have injured my body”) showed no improvement during the intervention, possibly since it could have been understood in the context of early post-surgery, when patients attribute acute pain to surgery-induced tissue damage. The findings of highest correlation at lag 0 (concurrent change) between pain intensity, pain catastrophizing and steps/day support the visual analysis. Previous studies report similar findings on temporal associations between process variables and functional outcomes among patients with chronic low back pain [57, 58], highlighting potential reciprocal effects between cognitive, emotional, and behavioral variables [58]. The explorative cross-lagged findings should be interpreted preliminarily given the study design. However, weekly measures provided additional insights into change patterns during the intervention.

### Treatment fidelity

Participation in the planned intervention sessions was high. In line with the person-centered approach, participant preferences were considered when the intervention period was planned. Participants often opted out of the second core session preoperatively due to their preferences and short times before surgery; the full-scale RCT could reinstate a structured, self-guided pain education component. Since weekly measurements indicated a flatter increase in steps per day around postoperative weeks 7–9, an additional session then may strengthen the intervention. Our study’s most frequently used BCTs align with those of previous studies, including interventions using BCTs to enhance physical activity adherence in populations with chronic musculoskeletal conditions [59]. Our study’s physical therapists seemingly followed a person-centered approach in securing their partnership with participants throughout the intervention. However, health plans’ documentation of adaptations should be clarified to involve patients as active partners in rehabilitation. Our findings regarding treatment fidelity suggest that the intervention was delivered largely as intended and can be used in a full-scale RCT with some modifications, particularly to strengthen the person-centered approach.

We did not pre-specify formal progression criteria. During study design and registration, there was insufficient prior evidence to meaningfully inform strict thresholds, consistent with the CONSORT extension for randomized pilot and feasibility trials [19]. Subsequent publications, such as the work of Mellor et al. [60], have emphasized that progression criteria should ideally be specified a priori to reduce the risk of optimism bias when judging feasibility. We interpreted our findings with this in mind and will implement several protocol refinements before proceeding to a full-scale RCT (Additional file 3).

## Conclusions

Overall, this study indicates that Get Back is feasible for patients undergoing decompression surgery due to central LSS, with modifications to strengthen the overall study procedure and the intervention before proceeding to a full-scale RCT. The following modifications are suggested:

- Revisit physical activity screening, considering eligibility rates and external validity.
- Redirect self-efficacy assessments to a more context-specific PROM.
- Reduce missing accelerometry data at follow-up using clearer instructions, automatic reminders, and address verification.
- Strengthen the structure regarding digital support for participants.
- Update sessions’ distribution.
- Revise and integrate the health plan and goal follow-up into the activity diary, enabling coherent co-creation and documentation.

## Supplementary Information

Additional file 1. Cross-lagged association between process variables and the outcome steps per day.

Additional file 2. CONSORT extension checklist.

Additional file 3. Progression Assessment by Domain.

## Data Availability

The data in the study are pseudonymized (coded) personal data, and Swedish legislation prohibits us from sharing the raw data. The datasets used and analyzed during the current study are available from the corresponding author upon reasonable request.

## Abbreviations

LSS: Lumbar spinal stenosis
WHO: World Health Organization
RCT: Randomized controlled trial
CONSORT: Consolidated Standards of Reporting Trials
TSK: Tampa Scale of Kinesiophobia
PCS: Pain Catastrophizing Scale
PROMs: Patient-Reported Outcome Measures
BCTs: Behavior Change Techniques
EQ-5D-3L: EuroQol 5-dimension 3-level version
EQ-5D VAS: EuroQol 5-dimension Visual Analog Scale
GSE: General Self-Efficacy
ODI: Oswestry Disability Index
NRS: Numeric Rating Scale
PCC: Person-Centered Care
SMA: Simulation Modeling Analysis
IQR: Inter-quartile range
BMI: Body Mass Index
CCI: Charlson Comorbidity Index
HADS-D: Depression subdomain of Hospital Anxiety and Depression Scale
ASA: American Society of Anesthesiologists
MVPA: Moderate to Vigorous Physical Activity
MCID: Minimal Clinically Important Difference
MIC: Minimally Important Change
