# Supplementary material for: Get Back, a person-centered digital program to promote physical activity among patients undergoing spinal stenosis surgery: a randomized feasibility study
Source: Pilot Feasibility Stud. 2026 Apr 24;12:55. doi: 10.1186/s40814-026-01826-6 (PMC13107828; doi:10.1186/s40814-026-01826-6)
Supplement: Supplementary file 1 — Additional file 1. Cross-lagged association between process variables and the outcome steps per day. [file 40814_2026_1826_MOESM1_ESM.docx]

**Additional table 1.** Cross-lagged association between process variables and the outcome steps per day.

|  | NRS | PCS Q8 | PCS Q5 | TSK Q7 | TSK Q12 | SE pain | SE PA | SE fall |
| --- | --- | --- | --- | --- | --- | --- | --- | --- |
| Lag: -2 | -.28,  *p* = .693 | -.71,  *p* = .405 | -.63,  *p* = .457 | .66,  ***p* = .005** | .37,  *p* = .065 | .08,  *p* = .140 | .26,  *p* = .071 | .26,  ***p* = .046** |
| Lag: -1 | -.13,  *p* = .599 | -.41,  *p* = .358 | -.37,  *p* = .393 | .67,  ***p* = .018** | .57,  ***p* = .037** | -.04,  *p* = .659 | .36,  *p* = .082 | -.03,  *p* = .712 |
| Lag: 0 | -.78,  ***p* = .003** | -.77,  ***p* = .007** | -.79,  ***p* = .008** | .20,  *p* = .300 | .58,  *p* = .057 | .44,  *p* = .104 | .56,  ***p* = .042** | .61,  ***p* = .018** |
| Lag: 1 | -.25,  *p* = .483 | -.77,  *p* = .130 | -.77,  *p* = .135 | .39,  *p* = .083 | .58,  ***p* = .037** | .36,  *p* = .099 | .45,  *p* = .056 | .19,  *p* = .141 |
| Lag: 2 | -.34,  *p* = .663 | -.26,  *p* = .686 | -.53,  *p* = .517 | .09,  *p* = .148 | .51,  ***p* = .027** | .45,  ***p* = .033** | .13,  *p* = .119 | .05,  *p* = .108 |

Notes: Values are Spearman Rho at Lags -5 to +5, where a positive lag indicates that changes in the process variables precedes changes in steps per day, and negative lags indicate the opposite, that changes in the process variables follows change in the steps per day. *p*-values are significant at < .05

NRS: Numeric rating scale, PCS Q8: Pain catastrophizing scale item 8, PCS Q5: Pain catastrophizing scale item 5, TSK Q7: Tampa scale of kinesiophobia item 7, TSK Q7: Tampa scale of kinesiophobia item 12, SE pain: pain-related self-efficacy, SE PA: self-efficacy for physical activity, SE fall: falls self-efficacy.
