# Supplementary material for: Get Back, a person-centered digital program to promote physical activity among patients undergoing spinal stenosis surgery: a randomized feasibility study
Source: Pilot Feasibility Stud. 2026 Apr 24;12:55. doi: 10.1186/s40814-026-01826-6 (PMC13107828; doi:10.1186/s40814-026-01826-6)
Supplement: Supplementary file 3 — Additional file 3. Progression Assessment by Domain. [file 40814_2026_1826_MOESM3_ESM.docx]

**Additional Table 3.** Progression Assessment by Domain

| **Research Question** | **Results** | **Interpretation** | **Progression Assessment to RCT** |
| --- | --- | --- | --- |
| **Domain: Process and Resource Feasibility** | | | |
| ***Research Question 1.1:*** What percentage of patients planned for decompression surgery for lumbar spinal stenosis (LSS) meeting inclusion criteria are eligible after the screening procedure? | 98/226 (43%) met the eligibility criteria for self-reported physical inactivity <150 min/week | The recruitment margin was limited, partly constrained by the physical inactivity criterion, which might affect the feasibility of a full-scale RCT | **Feasible with modifications:**   - Omit self-reported physical inactivity eligibility criterion since it may not capture the target population.   Rationale: Expected to enhance eligibility and recruitment for the main trial |
| ***Research Question 1.2:*** What are the reasons for declining participation in the study or dropping out? | 29/67 (43%) consented; reason for decline was reluctant to use digital format (33/38); drop out: after randomization, two surgeries were canceled, and two participants withdrew | The consent rate was acceptable, and the main barrier was identified and is addressable (digital hesitancy, rather than rejection of the intervention) with few dropouts | **Feasible with modifications:**   - Add digital onboarding support   Rationale: Expected to improve accessibility for digitally hesitant patients and thereby enhance recruitment |
| ***Research Question 1.3:*** Is the screening questionnaire measuring physical activity level able to detect patients planned for decompression surgery for LSS with a low level of physical activity compared to accelerometer data at baseline? | 3/25 (12%) classified as inactive through screening met ≥150 min/week MVPA | Acceptable screening method | **Progression assessment to main trial:** In relation to Research Question 1.1, this screening is not applicable. |
| ***Research Question 1.4*:** How many of the planned sessions of the Get Back_feasibility_ intervention do patients of the intervention group attend? | All participants (n=12) started Core Session 1; 8 of 12 omitted Core Session 2, and 4 of 12 replaced it with a phone call; all 12 continued post-operatively, completing a median of 4 booster sessions | High attendance shows that the multi-phase, remote intervention is feasible, and omitting or replacing the second pre-op session was due to scheduling, not low acceptability | **Feasible with modifications:**   - Redistribute Core Session 2   Rationale: Expected to make the intervention better align with patient preferences and surgical timelines |
| ***Research Question 1.5*:** Did the study participants and physical therapists in the study find the digital format, Get Back_feasibility_ intervention, and outcome measures relevant and usable? | Digital format: half of participants (6/12) reported that video sessions enabled visual contact and feedback, enhancing rehabilitation; the most common benefit was convenience, e.g., not having to travel (10/12 participants)  Relevance and usability: participants found the PROMs and digital capacity tests relevant and usable but burdensome with the number of PROMs | The digital format was generally acceptable and convenient, but the volume of PROMs reduced the perceived ease of participation | **Feasible with modifications:**   - Replace PROMs with validated short-form versions   Rationale: Expected to lower the participant burden without compromising outcome quality |
| ***Research Question 1.6*:** Is the Get Back_feasibility_ treatment safe (type and frequency of adverse events) in patients undergoing decompression surgery for LSS? | No adverse events were reported that were directly attributed to the intervention during the study | The intervention is safe for this postoperative population | **Feasible without modifications** |
| ***Research Question* 1.7:** What is the response rate of the PROMs and to what extent are physical tests completed in patients undergoing decompression surgery for LSS? If they are not completed, what are the reasons? | 100% response rate for PROMs and physical tests at baseline and follow-up; accelerometer completion was 92% at baseline and 76% at follow-up; reasons for missing accelerometer data: non-wearing, address-related delivery issues, and insufficient valid days | Retention was, overall, high and acceptable for accelerometer completeness, with potential for improvement | **Feasible with modifications:**   - Improve accelerometer instructions - Add automatic reminders - Verify current home addresses before posting the accelerometer   Rationale: Expected to improve accelerometer data collection. |
| **Domain: Outcomes related to intervention content** | | | |
| ***Research Question 2.1:*** Do the assessments preoperatively and at 12-week follow-up of steps per day, physical activity and pain catastrophizing, fear of movement and general self-efficacy provide tentative information as to the efficacy of the Get Back_feasibility_ intervention in patients undergoing decompression surgery for LSS? | The intervention group showed larger postoperative improvement in daily step counts than the control group; both groups improved their fear of movement, pain catastrophizing, and general self-efficacy | Steps per day were interpreted as promising as the definitive RCTs planned primary outcome. Relevance of general self-efficacy is difficult to interpret in relation to the intervention | **Suggested modifications:**   - Change to domain-specific self-efficacy (exercise/pain)   Rationale: Consider domain-specific self-efficacy (exercise/pain) to better capture self-efficacy relevant in the rehabilitation |
| ***Research Question 2.2:*** Do the weekly assessments of single-item questions aiming to measure steps per day, physical activity and aspects of pain catastrophizing, fear of movement and self-efficacy provide additional information regarding the efficacy of the Get Back_feasibility_ intervention, trajectories of change and interrelations between variables in patients undergoing decompression surgery for LSS? | There were large variations in all variables on individual level; at group level, steps first plateaued mid-treatment then increased in the later phase of the intervention period; psychological items stabilized early | Indicates the importance of a person-centered approach to the rehabilitation program with continued room for personalized adaptation in the treatment manual | **Suggested modifications:**   - Relocate core session to the plateau-phase |
| **Domain: Treatment Fidelity** | | | |
| ***Research Question 3.1:*** Is the treatment dose and content of Get Back_feasibility_ delivered as intended in patients undergoing decompression surgery for LSS? | All participants attended 4 core sessions; on average core session 1 lasted 47 minutes, and core sessions 3-5 32 minutes; a range of BCTs were frequently used | High fidelity to key components and flexibility around scheduling of core sessions when Session 2 was omitted reflects person-centered adaptation, rather than protocol failure | **Feasible with modifications:**   - Redistribute core session 2   Rationale: Expected to make the intervention better align with patient preferences and surgical timelines |
| ***Research Question 3.2:*** Does the physical therapist delivering the Get Back_feasibility_ intervention adhere to a person-centered approach? | Physical therapists reported full adherence to the GPCC framework routines; all participants reported feeling involved; qualitative data supported perceptions of being listened to, shared planning, and personal tailoring; some participants did not view the “health plan” as a separate document | Indications of a person-centered approach; the identified issue concerns the terminology and documentation of the health plan, not a lack of partnership | **Feasible with modifications:**   - Revise and integrate the health plan and goal follow-up into the activity diary   Rationale**:** Create one coherent, user-friendly tool for co-creation, documentation, and progress monitoring to secure the person-centered approach |

**Progression to full-scale RCT judged feasible with modifications.** In line with the CONSORT extension for pilot and feasibility trials and recommendations by Thabane et al., the findings indicate that a definitive randomized controlled trial is feasible with protocol modifications. Remaining issues (recruitment strategy, digital support, outcome burden, accelerometer procedures, preoperative scheduling, and documentation of the health plan) are judged remediable and do not warrant abandoning or fundamentally redesigning the intervention.
